# A Novel TetR Family Transcriptional Regulator, SAV576, Negatively Controls Avermectin Biosynthesis in *Streptomyces avermitilis*

**DOI:** 10.1371/journal.pone.0071330

**Published:** 2013-08-13

**Authors:** Jia Guo, Xuan Zhang, Shuai Luo, Fei He, Zhi Chen, Ying Wen, Jilun Li

**Affiliations:** State Key Laboratory of Agro-Biotechnology and MOA Key Laboratory of Soil Microbiology, College of Biological Sciences, China Agricultural University, Beijing, P. R. China; Centre National de la Recherche Scientifique, Aix-Marseille Université, France

## Abstract

Avermectins produced by *Streptomyces avermitilis* are potent anti-parasitic agents that are useful in animal health care, agriculture, and the treatment of human infections. In a search for novel regulators that affect avermectin biosynthesis, comparative transcriptome analysis was performed between wild-type strain ATCC31267 and avermectin overproducing strain 76-02-e, revealing some differentially expressed genes. *SAV576*, which is downregulated in 76-02-e and encodes a TetR family transcriptional regulator (TFR), was shown to inhibit avermectin production by indirectly affecting the expression of *ave* genes. SAV576 directly repressed the transcription of its gene *SAV576* and of adjacent genes *SAV575* (encodes cytochrome P450/NADPH-ferrihemoprotein reductase) and *SAV574*. The SAV576-binding sites within the bidirectional *SAV575*-*SAV576* promoter region were determined by DNase I footprinting assays. A consensus 15-bp palindromic sequence CCRTACRVYGTATGS was found in these binding sites and shown to be important for SAV576-binding activity. *SAV575*, an important target gene of SAV576, was shown to exert a positive effect on avermectin production. The study findings extend our limited knowledge of the complex regulation of avermectin biosynthesis and provide a basis for rational genetic manipulation of *S. avermitilis* to improve avermectin production through control of SAV576 and its target gene.

## Introduction

*Streptomyces* species are gram-positive filamentous soil bacteria known for their ability to produce a wide range of bioactive secondary metabolites during their complex life cycle. These metabolites include many useful antibiotics that display antibacterial, anticancer, anthelmintic, and/or immunosuppressive activities [1], [2]. The genes responsible for the biosynthesis of these antibiotics are usually clustered, and are co-regulated by pathway-specific regulatory genes and various higher-level pleiotropic regulatory genes [3]. The initiation of the expression of these regulatory genes is affected by a variety of environmental and physiological factors, including growth rate, imbalances in metabolism [4], nutrient levels (carbon, nitrogen, and phosphate) [5]–[10], and small signaling molecules such as γ-butyrolactone [11]–[13] and ppGpp [14]–[16]. The production of the antibiotics is thus a complex process that is tightly regulated at multiple genetic levels.

Avermectins are a series of potent anthelmintic and insecticidal macrolide antibiotics (A1a, A1b, A2a, A2b, B1a, B1b, B2a, and B2b) produced by *S. avermitilis*. They are used commercially for broad-spectrum parasite control in medical, veterinary, and agricultural fields [17], [18]. The avermectin biosynthetic pathway has been well elucidated [18]–[20], and the complete *S. avermitilis* genome has been sequenced [21], [22]; however, the complex regulatory mechanisms of avermectin production remain poorly understood. The regulatory genes that are reportedly involved in avermectin biosynthesis include: *aveR* [19], [23]–[25], *aveR1/aveR2* [26], *orfX* [27], *afsK-av* [28], *aveI* [29], [30], *SAV3818* [31] and *avaR3* [32]. Further studies are required to identify other yet-unknown regulatory genes, which will contribute to better understanding of the regulatory networks of avermectin biosynthesis and to the practical construction of avermectin high-producing strains.

Comparative transcriptome analysis has been applied increasingly during the past decade to identify alterations of gene expression in antibiotic-overproducing *Streptomyces* strains [33]–[35] and has been shown to be an efficient technique for the discovery of novel regulatory genes. In the present study, we compared the transcriptomes of *S. avermitilis* wild-type strain ATCC31267 and avermectin high-producer 76-02-e using a *S. avermitilis* whole-genome microarray chip and thereby revealed some putative regulatory genes that may be related to avermectin biosynthesis. We further characterized a previously unknown TetR family transcriptional regulator (TFR) gene, *SAV576*, as an important avermectin downregulator, and demonstrated that SAV576 inhibits avermectin production by modulating the transcription level of its target genes and *ave* genes.

## Materials and Methods

### Strains, Plasmids, and Growth Conditions

*S. avermitilis* wild-type strain ATCC31267 was used as a host strain for gene propagation and gene disruption. 76-02-e, an avermectin high-producer, was derived from ATCC31267 by continuous mutagenesis (two rounds of high frequency electronic flow mutagenesis, five rounds of NTG mutagenesis, Co^60^ mutagenesis, and three rounds of UV mutagenesis) and was collected in our laboratory [36]. *S. avermitilis* strains were grown at 28°C on solid YMS medium [6] for sporulation or in liquid YEME medium [37] containing 25% sucrose for growth of mycelia. RM14 medium [38] was used for regeneration of protoplasts and for selection of transformants. MM agar [37] was used for observation of *S. avermitilis* phenotype. Seed medium and fermentation medium FM-I used for avermectin production were as described previously [39]. Because FM-I contains insoluble yeast meal, soluble fermentation medium FM-II [25] was used to cultivate mycelia for growth and ChIP analysis and for RNA isolation. In comparison to ATCC31267, 76-02-e produced amounts of avermectins that were approximately 40-fold higher (∼5000 µg/ml) when grown in FM-I, and 5-fold higher (∼600 µg/ml) when grown in FM-II.

*E. coli* strains JM109 and BL21 (DE3) (Novagen) were used as the cloning host and the expression host, respectively. *E. coli* ET12567 (*dam dcm hsdS*) [38] was used to propagate non-methylated DNA for transformation into *S. avermitilis*. *E. coli* strains were grown at 37°C in Luria-Bertani (LB) medium [40]. The antibiotics used were described previously [41]. Multiple-copy vector pKC1139 [42] was used for gene disruption and overexpression in *S. avermitilis*. pSET152 [42] was used to introduce a single-copy gene into *S. avermitilis*. pET-28a (+) (Novagen) was used for production of recombinant His_6_-tagged protein in *E. coli*.

### Microarray Assays

Mycelia of ATCC31267 or 76-02-e grown in FM-II were collected on days 2 and 6, ﬂash-frozen in liquid nitrogen, and ground into a fine powder. RNA was extracted using Trizol reagent (Tiangen, China) according to the manufacturer’s instructions. Agilent microarrays (8×15K) for the analysis of *S. avermitilis* gene expression were designed and manufactured by Shanghai Biochip Co. Ltd (SBC, China), based on publicly available complete genome sequence information (http://avermitilis.ls.kitasato-u.ac.jp). For each gene, two different 60-mer oligonucleotides were designed. Each slide contained a total of 7670 open reading frames (ORFs). The microarray assays, including labeling, hybridization, washing, and microarray data normalization, were performed by SBC.

### Microarray Dataset Accession Number

The raw microarray dataset used in this study has been submitted to NCBI Gene Expression Omnibus under the accession number GSE47223.

### Gene Disruption, Complementation, and Overexpression

**Figure 1:**
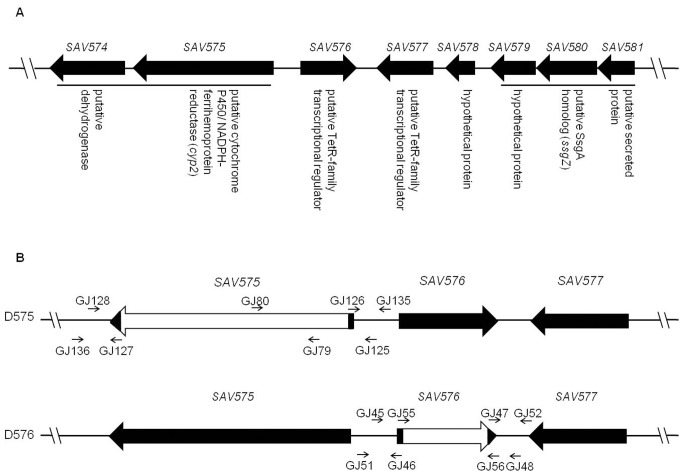
Organization of *SAV576* and its adjacent genes on the chromosome of *S. avermitilis* (A) and schematic representation of the strategy used for deletion of *SAV575* and *SAV576* genes (B). (**A**) Gene notations are based on the Genome Project of *S. avermitilis* (http://avermitilis.ls.kitasato-u.ac.jp/). The two transcriptional units are indicated by black bars. (**B**) Long black arrows indicate genes and their directions. Short arrows indicate the positions of primers used for cloning exchange regions and confirming gene deletions. White blocks represent in-frame deletions in the corresponding genes.

To construct a *SAV576* deletion mutant, two fragments flanking *SAV576* were prepared by PCR from the genomic DNA of ATCC31267. A 796-bp 5′ flanking region was amplified with primers GJ45 and GJ46, and a 746-bp 3′ flanking region was amplified with primers GJ47 and GJ48. The two PCR fragments were ligated into pKC1139 to generate a *SAV576*-deletion vector pDGJ576. Transformation of pDGJ576 into ATCC31267 and selection of double-crossover recombinant strains were performed as described previously [41]. The *SAV576*-deleted mutant D576 was confirmed by PCR using primers GJ51, GJ52, GJ55, and GJ56 (Fig. 1B). When primers GJ51 and GJ52, which flank the exchange regions, were used for PCR analysis of putative *SAV576* deletion mutant, a 1.75-kb band appeared, whereas a 2.36-kb band was detected when genomic DNA of ATCC31267 was used as the template. When using primers GJ55 and GJ56 located within the deletion region of *SAV576* gene, only ATCC31267 produced a 0.9-kb PCR fragment as predicted (data not shown). For complementation of D576, a 1.57-kb DNA fragment carrying the *SAV576* ORF and its putative promoter was amplified by PCR with primers GJ45 and GJ56*. The PCR product was inserted into pSET152 to generate *SAV576* complementation vector pSET152-576, which was then introduced into D576 to obtain the complemented strain. The 1.57-kb *Eco*RI/*Xba*I fragment containing the *SAV576* from pSET152-576 was cloned into pKC1139 to produce pKC1139-576, which was used for overexpession of *SAV576* in ATCC31267.

To construct a *SAV575* deletion mutant, a 739-bp 5′ flanking region and a 517-bp 3′ flanking region were amplified with primer pairs GJ125/GJ126 and GJ127/GJ128, respectively. The two PCR fragments were cloned into pKC1139 to produce a *SAV575*-deletion vector pDGJ575, which was then introduced into ATCC31267. The resulting *SAV575*-deleted mutant D575 was confirmed by PCR using primers GJ135, GJ136, GJ79, and GJ80 (Fig. 1B). When using primers GJ135 and GJ136, which flank the exchange regions, a 1.48-kb band appeared, whereas the theoretical 4.46-kb band was too large to be detected when genomic DNA of ATCC31267 was used as the template. When using primers GJ79 and GJ80 located within the deletion region of *SAV575*, only ATCC31267 yielded a 369-bp PCR fragment (data not shown). A 4.13-kb DNA fragment carrying the promoter and the coding region of *SAV575* was amplified with primers GJ89 and GJ90, and was cloned into pKC1139 to give pKC1139-575 for overexpression of *SAV575* in ATCC31267 or in D576.

The construction of *SAV575-576* double mutant D575-576 was similar to that of D575, using D576 as host strain. All primers used in this study are listed in Table S1.

### Semiquantitative and Real-time RT-PCR Analyses

RNA extractions were carried out with Trizol (Tiangen) from cultures of *S. avermitilis* at various times. To remove chromosomal DNA contamination, each RNA sample was treated with DNase I and tested by PCR to confirm the absence of chromosomal DNA. The treated RNA sample (2 µg) was reverse transcribed using M-MLV (RNase H^-^, TaKaRa), random hexamers (25 µM), and a dNTP mixture (10 mM each). Semiquantitative RT-PCR analysis was performed to determine the transcription levels of various genes using the obtained cDNA as template and the primers listed in Table S1. The *hrdB* gene, which encodes the major sigma factor in *Streptomyces*, was used as a positive internal control in the RT-PCR assays. The obtained cDNAs were also used as templates for real-time PCR analysis. The experiments were performed using FastStart Universal SYBR Green Master (ROX) (Roche) with analysis by an ABI 7900HI Sequence Detection System using optical-grade 96-well plates. Template cDNA, 10 µl FastStart Universal SYBR Green Master (ROX), and forward and reverse primers (each 300 nM) were mixed in each reaction system (total volume 20 µl). The PCR protocol consisted of 95°C for 10 min, 40 cycles of 95°C for 10 s, and 60°C for 30 s with a single ﬂuorescence measurement.

### Overexpression and Purification of Recombinant His6-tagged SAV576

A DNA fragment encoding the predicted 218 amino acids of SAV576 protein was generated by PCR with primers GJ71 and GJ72. The PCR fragment was inserted into the expression vector pET-28a (+) to generate pET28-576, which was then introduced into *E. coli* BL21 (DE3) for protein overexpression. Following induction by IPTG, the resulting recombinant His_6_-tagged SAV576 protein was purified on a Ni^2+^-NTA spin column according to the manufacturer’s instructions (Qiagen). The purified protein was used for antibody induction, EMSA, and DNase I footprinting assays.

### Preparation of Antibodies against SAV576 Protein

Polyclonal antibodies against SAV576 were prepared by Beijing Protein Innovation (China). 200 µg purified recombinant protein His_6_-SAV576 was mixed with Freund’s complete adjuvant and injected into a rabbit. After 2 weeks, the antigen was injected into the same rabbit with Freund’s incomplete adjuvant. Further booster immunizations were given at 11-day intervals. The rabbit was bled 10 days after each boost, and serum was prepared. Each serum was stored at 4°C, and its potency was checked by ELISA. After three booster immunizations, the immune serum attained a high potency and was used as a source of anti-SAV576 antibodies.

### Chromatin Immunoprecipitation (ChIP) Assay

The ChIP protocol was as described previously [25]. Briefly, *S. avermitilis* cultures grown in FM-II for 72 h were fixed in cross-linked buffer [0.4 M sucrose, 10 mM Tris·Cl (pH 8.0), 1 mM EDTA] containing 1% formaldehyde for 20 min at 28°C. Chromatin immunoprecipitation was performed using anti-SAV576 antibody. After DNA extraction, pellets were washed with 70% ethanol and resuspended in 50 µl TE, and 2 µl DNA solution was used for PCR using the primer sets listed in Table S1.

### Western Blotting

Western blotting analyses were performed as described previously [25]. Polyclonal antibody against AveR [25] or SAV576 was used at a dilution of 1∶1,000. Western blots were developed with polyclonal antibodies using an ECL detection system (Roche).

### Electrophoretic Mobility Shift Assay (EMSA)

EMSAs were performed using a DIG Gel Shift Kit, 2^nd^ Generation (Roche). The probes were amplified by PCR and labeled at the 3′ ends with nonradioactive digoxigenin (DIG). The 20 µl reaction mixture contained the probes, proteins, and 1 µg poly [d(I-C)] in a binding buffer. The mixture was incubated at 25°C for 30 min and then added with 5 µl loading buffer with bromophenol blue. Protein-bound and free DNA were separated by electrophoresis on non-denaturing 5% polyacrylamide gels with 0.5×TBE as running buffer, and were then transferred onto nylon membranes by electroblotting. The membranes were baked for 10 min at 80°C, and the DNA fragments were cross-linked by exposure to UV radiation for 10 min. Chemiluminescence detection was performed according to the manufacturer’s instructions, and the membranes were exposed to X-ray film (Fuji) for 15–30 min.

### DNase I Footprinting

A non-radiochemical capillary electrophoresis method was used for DNase I footprinting [43]. To characterize the binding sites of SAV576 protein in the SAV575-SAV576 intergenic region, two fluorescence-labeled DNA fragments were synthesized by PCR using primer pairs FAM-GJ78/GJ77 and FAM-GJ228/GJ227. The resulting 547-bp and 478-bp DNA fragments covered the entire intergenic region. Following purification, labeled DNA fragments (400 ng) and appropriate concentrations of His_6_-tagged SAV576 protein were added to a final reaction volume of 50 µl and incubated for 30 min at 25°C. Digestion with DNase I (0.016 units) was performed for 40 s at 37°C and stopped by the addition of EDTA at a final concentration of 60 mM. The reaction mixture was heated to 80°C for 10 min to totally inactivate DNase I. The samples were subjected to phenol-chloroform extraction, ethanol precipitation, and capillary electrophoresis by loading into an Applied Biosystems 3730 DNA Genetic Analyser together with the internal-lane size standard ROX-500 (Applied Biosystems). Electrophoregrams were analyzed using the GeneMarker program, v1.8 (Applied Biosystems).

### Identification of the Transcriptional Start Point Using 5′-RACE

To determine the transcriptional start point of *SAV575* and *SAV576*, total RNA was extracted from an 84-h culture of ATCC31267 grown on YMS medium. 2 µg total RNA was used for reverse transcription with 20 pmol of gene-specific primer 575SP1 or 576SP1 using a 5′/3′ RACE Kit (2^nd^ Generation, Roche). The sample was purified using a PCR Product Purification Kit (Beijing HT-Biotech Co. Ltd, China). An oligo-dA tail was added to the 3′ end of the cDNA using terminal deoxynucleotidyl transferase, followed by direct amplification of the tailed cDNA using the oligo dT-anchor primer (GACCACGCGTATCGATGTCGACTTTTTTTTTTTTTTTTV) and a second inner gene-specific primer 575SP2 or 576SP2. An additional round of PCR was performed with a 1,000-fold dilution of the original PCR product as template, using an anchor primer (GACCACGCGTATCGATGTCGAC) and a nested 575SP3 or 576SP3 primer, to obtain a single specific band. The final PCR products were cloned into pMD18-T vector (TaKaRa) for sequencing. The first nucleotide following the oligo-dA sequence was considered to be the transcriptional start point.

### Fermentation and HPLC Analysis of Avermectin Production

Fermentation of *S. avermitilis* ATCC31267 and its mutants was performed, and avermectins in the fermentation culture were identified by HPLC analysis as described previously [39].

## Results

### Transcriptome Comparison between Wild-type and Avermectin Overproducing Strains

Total RNA was isolated from mycelia of *S. avermitilis* wild-type strain ATCC31267 and avermectin overproducing strain 76-02-e grown in FM-II at day 2 (early exponential phase) and day 6 (stationary phase), and was used for microarray assays. Comparative transcriptome analysis revealed that 162 genes were upregulated and 150 were downregulated 2-fold or more in 76-02-e relative to ATCC31267 at both of the studied time points. Most of the differentially expressed genes encoded unknown or unclassified proteins (upregulated 73, downregulated 85). Smaller numbers of genes were involved in regulatory functions (upregulated 16, downregulated 16), transport and binding proteins (upregulated 11, downregulated 7), mobile and extrachromosomal element functions (upregulated 14, downregulated 6), or secondary metabolism (upregulated 20, downregulated 18). The remaining genes were associated with sig-regulons, amino acid metabolism, fatty acid and phospholipid metabolism, carbohydrate metabolism, purine/pyrimidine metabolism, biosynthesis of cofactors and prosthetic groups, protein synthesis, folding and modification, RNA recombination, cell envelope, cell division/morphological differentiation, or gas vesicle cluster (File S1). As expected, most of the genes (17 of 19) in the avermectin biosynthetic gene cluster, including *aveR* (encoding a pathway-specific activator) and *aveA1* (encoding polyketide synthase AVES1), were significantly overexpressed in 76-02-e (File S1), which was consistent with the increased avermectin production.

Real-time RT-PCR was used to test five upregulated genes (*aveR*, *aveA1*, *SAV292*, *SAV880*, *SAV4189*) and two downregulated genes (*SAV576*, *SAV151*). The PCR results were mainly consistent with the microarray data (Fig. S1), demonstrating the general reliability of the microarray data. Among the tested genes, *SAV292*, *SAV880*, *SAV576* and *SAV151* encode TFRs, and *SAV4189* encodes a MarR-family transcriptional regulator. The functions of these regulatory genes were not determined. The *SAV576* gene was of particular interest. The microarray data indicated that *SAV576* in 76-02-e was downregulated 37-fold at day 2 and 93-fold at day 6 (File S1), suggesting that this gene is involved in the regulation of avermectin biosynthesis. We therefore performed functional analysis of *SAV576*.

### Characterization of *SAV576* and Its Adjacent Genes

The *SAV576* gene, located in the left arm of the chromosome, contains 657 nucleotides and encodes a TFR of 218 amino acids, including a common N-terminal helix-turn-helix DNA-binding domain and a C-terminal all-alpha domain homologous to TetR. The divergently transcribed genes *SAV575* and *SAV574* are located upstream of *SAV576*. *SAV575* (*cyp2*) encodes a putative cytochrome P450/NADPH-ferrihemoprotein reductase, and *SAV574* encodes a putative dehydrogenase (Fig. 1A). *SAV577* is another TFR gene located downstream of *SAV576*. *SAV578* and *SAV579* both encode hypothetical proteins. *SAV580* (*ssgZ*) is an *ssgA* homologous gene, and *SAV581* encodes a putative secreted protein. RT-PCR analysis using primers that amplify intergenic regions revealed that *SAV574* and *SAV575* are co-transcribed, and that *SAV579-SAV580-SAV581* forms another transcriptional unit (Fig. S2). The *SAV576*, *SAV577*, *SAV578*, *SAV579*, and *SAV581* genes are specific to *S. avermitilis* and have no orthologs in other sequenced *Streptomyces* genomes.

### SAV576 Plays an Important Role in Avermectin Production

**Figure 2:**
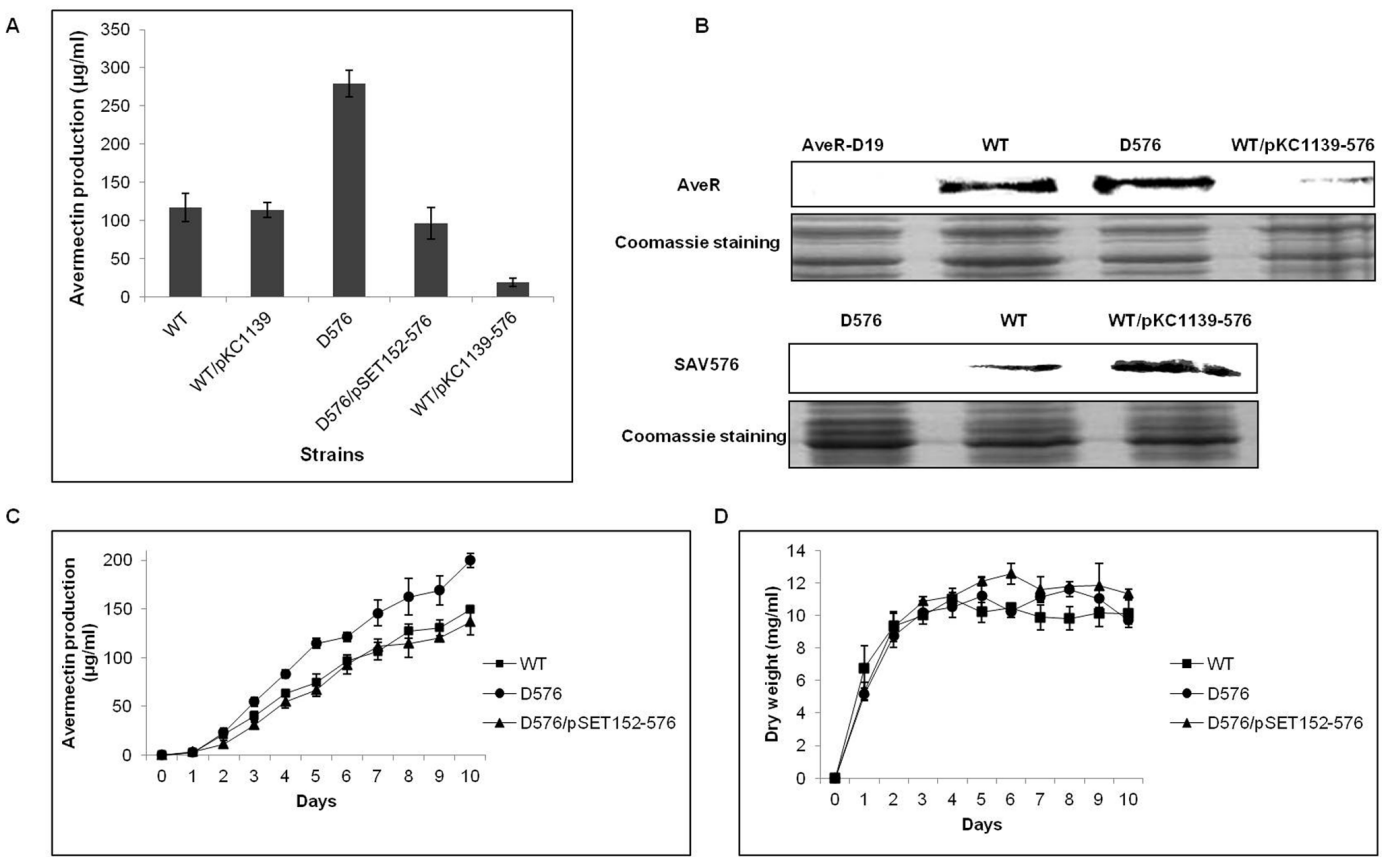
Avermectin production and growth of wild-type ATCC31267 and *SAV576* mutant strains. (**A**) Comparison of avermectin production in various *S. avermitilis* strains grown in FM-I medium for 10 days. WT, wild-type strain ATCC31267; WT/pKC1139, ATCC31267 carrying control plasmid pKC1139; D576, *SAV576* deletion mutant; D576/pSET152-576, complementation strain of D576; WT/pKC1139-576, *SAV576* overexpression strain. (**B**) Western blotting analysis of SAV576 and AveR protein in cells grown in FM-I for 6 days. Approximately 100 µg total protein of each sample was subjected to Western blot and Coomassie Blue staining for the loading control. AveR-D19, *aveR* mutant. (**C** and **D**) Effect of *SAV576* deletion on avermectin production (**C**) and growth (**D**) of *S. avermitilis* grown in FM-II. Solid squares, ATCC31267; Solid circles, D576; Solid triangles, complementation strain of D576.

To assess the role of *SAV576* in *S. avermitilis*, the *SAV576* deletion mutant D576 was constructed (Fig. 1B). D576 displayed no obvious differences from the wild-type strain when grown on YMS, RM14, or MM solid medium (data not shown). HPLC analysis of the fermentation products following cultivation in FM-I for 10 days showed that avermectin production in D576 was ∼2.3-fold compared to that in ATCC31267 (Fig. 2A). Avermectin production was restored in the *SAV576* gene complementation strain (D576/pSET152-576), confirming that the increased avermectin production in D576 was due solely to the deletion of *SAV576*. The enhancement of *SAV576* expression in wild-type strain (WT/pKC1139-576) led to a clear reduction in avermectin yield (Fig. 2A). The deletion and overexpression of *SAV576* in wild-type strain were confirmed by Western blotting (Fig. 2B). Western blotting analysis of the avermectin pathway-specific activator AveR showed that, in agreement with the avermectin yield, AveR was overexpressed in D576 but underexpressed in WT/pKC1139-576 (Fig. 2B). These findings suggest that *SAV576* inhibits avermectin production by directly or indirectly affecting the expression of *aveR*.

To investigate whether the avermectin overproduction in D576 was due to increased cell growth, we analyzed the growth and avermectin production of ATCC31267, D576, and D576/pSET152-576 cultured in FM-II. The deletion of *SAV576* resulted in increased avermectin production (Fig. 2C), but did not affect cell growth (Fig. 2D). The growth and avermectin production of D576/pSET152-576 were both similar to those of ATCC31267. These findings indicate that *SAV576* acts to inhibit avermectin biosynthesis, but has no effect on cell growth.

### The Transcription Levels of SAV576 Vary at Different Life Stages

**Figure 3:**
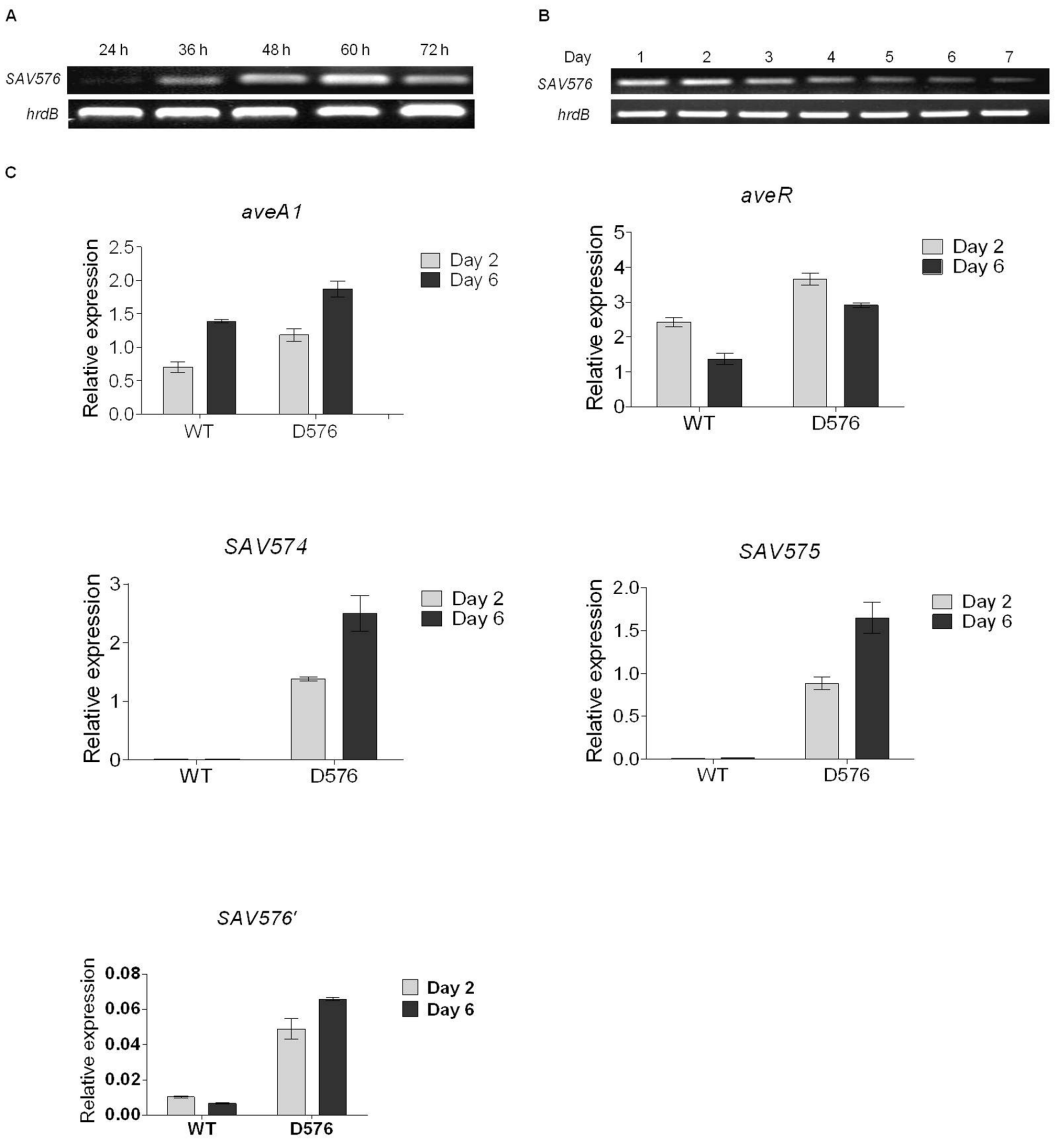
Transcriptional analysis of *SAV576* and related genes. (**A** and **B**) Semiquantitative RT-PCR analysis of transcription levels of *SAV576* in ATCC31267 grown on solid medium YMS (**A**) and in liquid medium FM-II (**B**) for various durations. The 214-bp *SAV576* transcript was amplified from the internal coding region of *SAV576* with primers GJ83 and GJ84. *hrdB* was used as a positive internal control. (**C**) Real-time RT-PCR analysis of *aveR*, *aveA1*, *SAV574*, *SAV575* and *SAV576* transcription levels from ATCC31267 (WT) and D576 grown in FM-II on days 2 and 6. Relative values were obtained using *hrdB* as a reference. *SAV576′*, 142-bp transcript amplified from the *SAV576* promoter region and the remainder ORF of D576 with primers GJ46* and GJ55*.

To determine the transcription levels of *SAV576* at various life stages, semiquantitative RT-PCR was performed using RNA isolated from ATCC31267 grown on solid sporulation medium YMS, or in liquid fermentation medium FM-II for various durations. Various stages of *S. avermitilis* development were clearly observable on YMS: vegetative (substrate mycelia) growth (represented by the 24 h RNA sample) was followed by aerial growth (the 36-h, 48-h and 60-h RNA samples) and then by sporulation (>60 h, represented by the 72-h RNA sample). *SAV576* transcription was detectable throughout the life cycle, but its level varied depending on the time point. The transcription level was low at 24 h when the strain was growing as substrate mycelia, increased during aerial hyphae growth with a peak at 60 h, and then decreased at 72 h, when mature spores were evident (Fig. 3A). These data suggest that the transcription of *SAV576* is controlled by some factor(s) related to morphological development.

In the liquid medium FM-II, *S. avermitilis* was unable to sporulate but produced larger amount of avermectins than it did on solid medium. Avermectin production was not observable by HPLC on day 1, but then increased gradually from day 2 onward (Fig. 2C). RT-PCR analysis showed that *SAV576* transcription reached its maximal level on day 2 and then gradually declined (Fig. 3B). This finding is consistent with the inhibitory role of *SAV576* in avermectin biosynthesis.

### Transcription of Certain Genes is Affected by SAV576 Deletion

To find potential targets regulated by *SAV576*, RT-PCR was performed using RNAs isolated from ATCC31267 and from D576 grown for 2 or 6 days in FM-II, corresponding to the stages of the microarray data. Real-time RT-PCR analysis showed that both *aveR* and *aveA1* were upregulated in D576 (Fig. 3C), indicating that *SAV576* affects avermectin production by downregulating the expression of *ave* genes.

The transcription levels of *SAV576* and its adjacent divergently transcribed genes *SAV574* and *SAV575* were tested using the same RNA preparations. Each of these genes was significantly upregulated in D576 on both days (Fig. 3C), indicating that the *SAV576* gene product acts either directly or indirectly to downregulate the transcription of its own gene and of the adjacent genes.

### Binding of SAV576 to the Intergenic Region between *SAV575* and *SAV576*

TFRs are a common class of transcriptional factors that regulate target genes by binding to their promoters. To assess whether the regulation of the genes listed in the preceding section by SAV576 was direct, we performed ChIP assays and EMSAs.

**Figure 4:**
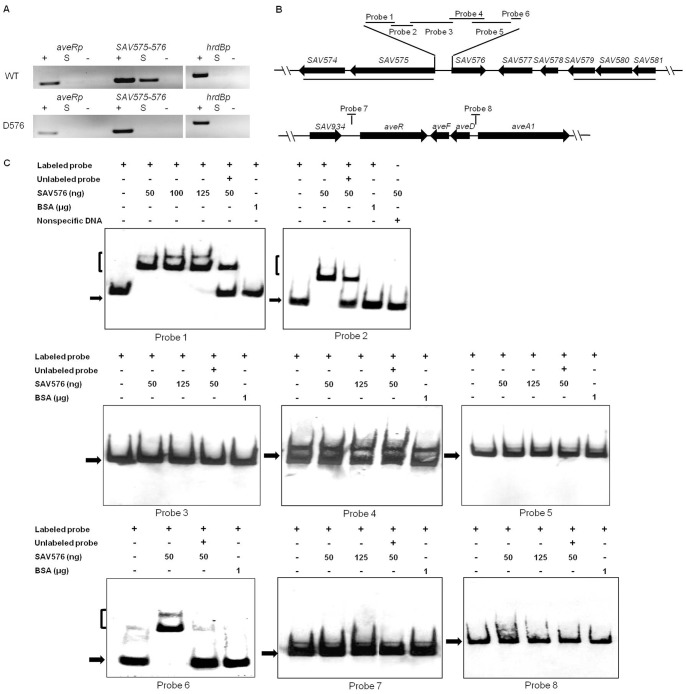
Analysis of SAV576 protein binding to target promoter regions. (**A**) ChIP assays *in* *vivo*. Anti-SAV576 antibodies were used to immunoprecipitate SAV576-DNA complexes from ATCC31267 and D576 cells treated with formaldehyde. The DNAs used for PCR were total DNA prior to immunoprecipitation (positive control: lanes “+”), immunoprecipitated DNA (experimental sample: lanes “S”), and negative control DNA without antibody (lanes “−”). The *hrdB* promoter region was used as a control. (**B**) Schematic representation of the relative positions of probes used for EMSAs *in* *vitro*. Probe 1, 98-bp DNA fragment from +2 to −96 relative to the translational start codon of *SAV575*; probe 2, 43-bp fragment from −98 to −140; probe 3, 276-bp fragment from −118 to −393; probe 4, 257-bp fragment from −306 to −562; probe 5, 333-bp DNA fragment from −533 to −865; probe 6, 43-bp DNA fragment from −866 to −908. Probe 7, 200-bp DNA fragment from −116 to −315 relative to the start codon of *aveR*. Probe 8, 328-bp DNA fragment from −14 to −341 relative to the start codon of *aveA1*. Probes 7 and 8 cover the putative transcriptional start points of *aveR* and *aveA1*, respectively. (**C**) EMSAs of the interaction of the probes with purified His_6_-SAV576 protein. Each lane contained 0.3 nM labeled probe. The labeled probe and an approximately 100-fold excess of the unlabeled probe were used in competitive assays. BSA was used as a negative control for SAV576 protein. Labeled non-specific DNA was used to eliminate non-specific binding of SAV576 protein. The free probes are indicated by solid arrows, and the retarded DNA fragments are indicated by parentheses.

ChIP assays are widely used to determine where DNA-binding proteins bind to the genome *in vivo*. Following 48 h growth in FM-II, avermectin production in D576 was higher than that in ATCC31267 (Fig. 2C), suggesting that the SAV576 protein binds to its DNA targets and thereby downregulates avermectin biosynthesis prior to this time point. ChIP assays were performed using *S. avermitilis* strains treated with formaldehyde at 72 h to cross-link the SAV576 protein to its DNA targets. The cross-linked DNA was extracted and fragmented by sonication, and immunoprecipitation was then performed using anti-SAV576 antibodies to screen the DNA fragments that were attached to the SAV576 protein. Two putative promoter regions were chosen on the basis of sequence analysis: a 200-bp region upstream of *aveR* and a 207-bp region at the *SAV575-SAV576* intergenic region containing two divergent promoters. The primer pairs used for PCR detection of the two promoter regions are listed in Table S1. All of the PCR bands of correct size were obtained from the positive control DNA of ATCC31267 or D576, whereas no such bands were detected when the negative control DNA without antibody was used as template (Fig. 4A). In comparison with the control *hrdB* promoter, it was clear that only the PCR product of the *SAV575-SAV576* intergenic region was selectively enriched when the immunoprecipitated DNA of ATCC31267 was used as template. In contrast, no correct PCR bands were amplified from the immunoprecipitated DNA of D576 (Fig. 4A). These results indicate that the SAV576 protein binds specifically to the bidirectional *SAV575-SAV576* promoter region, but not to the *aveR* promoter region *in vivo*.

To confirm that SAV576 protein binds directly to the above target promoters, we performed *in vitro* EMSAs using a full-length recombinant His_6_-SAV576 protein expressed in *E. coli*. The entire intergenic region between *SAV575* and *SAV576* was 900-bp in length, and six probes (designated probes 1, 2, 3, 4, 5, and 6) labeled with DIG were designed to cover this region. The 200-bp *aveR* promoter region used in the ChIP assays and the 327-bp *aveD*-*aveA1* intergenic region were labeled as probes 7 and 8, respectively (Fig. 4B). Results showed that the His_6_-SAV576 protein clearly retarded probes 1, 2, and 6, but not probes 3, 4, 5, 7, or 8 (Fig. 4C). A labeled nonspecific DNA probe and BSA were used as negative controls. These findings indicate that the SAV576 protein binds to the bidirectional *SAV575-SAV576* promoter region directly by binding DNA sites located within the sequences of probes 1, 2, and 6. These *in vitro* EMSA results are in agreement with those from the *in vivo* ChIP assays, indicating that the SAV576 protein indirectly regulates avermectin biosynthesis, but directly controls the transcription of its own gene and adjacent gene *SAV575* through interaction with their promoter regions. *SAV574* is co-transcribed with *SAV575* (Fig. S2), indicating that it is also a target gene of SAV576.

### Determination of the Binding Sites of the SAV576 Protein

**Figure 5:**
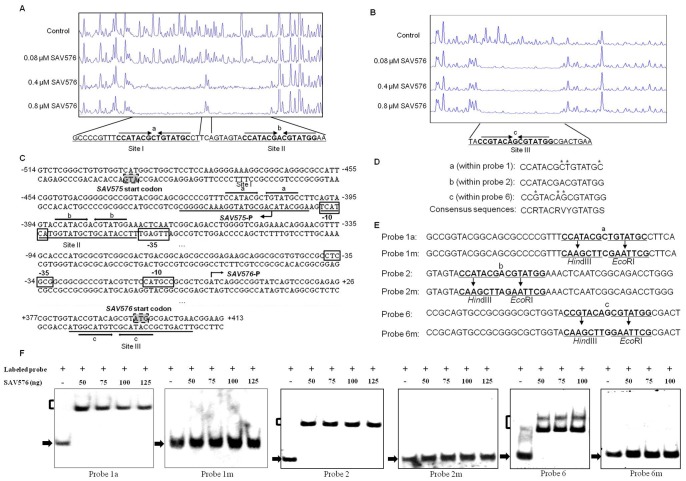
Determination of the binding sites of the SAV576 protein. (**A** and **B**) DNase I footprinting assay of SAV576 on the *SAV575* (**A**) and *SAV576* (**B**) promoter regions, respectively. The fluorograms correspond to the control DNA (10 µM BSA) and to the protection reactions with increasing concentrations of His_6_-SAV576 protein, respectively. (**C**) Nucleotide sequence of the *SAV575*-*SAV576* promoter region and SAV576-binding sites. The numbers indicate the distance (nt) from the transcriptional start point of *SAV576*. Solid lines, SAV576-binding sites; arrows, inverted repeats; bent arrows, transcriptional start points and transcription orientation; boxed areas, putative −10 and −35 regions; shaded areas, translational start codon. (**D**) Three 15-bp palindromic sequences “a”, “b”, and “c”. The mismatched nucleotides in comparison with sequence b are indicated by asterisks. (**E**) Mutations introduced into the 15-bp palindromic sequences. Each of the probes used was 43-bp. Probes 1a, 2, and 6 contained sequences a, b, and c, respectively. *Hin*dIII and *Eco*RI sites were generated at sequences a, b, and c to produce mutated probes 1m, 2m, and 6m, respectively. The nucleotides changed are indicated by underlining. (**F**) EMSAs using the mutated DNA probes. The free probes are indicated by solid arrows, and the retarded DNA fragments are indicated by parentheses.

The results of the transcription experiments and the EMSAs suggest that SAV576 regulates its own gene and *SAV575* by direct binding to three regions: probes 1, 2, and 6. To clarify the regulation mechanism of SAV576, we determined the promoter structures of *SAV575* and *SAV576* and the specific binding sites of SAV576 on the *SAV575-SAV576* intergenic region. The transcriptional start point (tsp) of *SAV575* was localized by 5′ RACE to C at position 87 nt upstream of the translational start codon of *SAV575* (Fig. 5C and S3A). The *SAV576* tsp was localized to A at position 405 nt upstream of the translational start codon of *SAV576* (Fig. 5C and S3B).

DNase I footprinting assays of the bidirectional *SAV575-SAV576* promoter region were performed in the presence and absence of His_6_-SAV576 protein, using a capillary sequencer to analyze the protected regions. As expected, three protected regions (sites I, II, and III) were identified on the coding strand of *SAV576* (Fig. 5A and B). These three sites were located respectively within the sequences of probes 1, 2, and 6, confirming the direct interaction of SAV576 with these probes. Site I extends from positions +19 to −7, and site II extends from positions −10 to −33, relative to the *SAV575* tsp. Sites I and II are separated by 2 nt, and site II overlaps the potential −10 region of the *SAV575* promoter (Fig. 5C), indicating that SAV576 negatively regulates *SAV575* transcription by blocking the access of RNA polymerase to its promoter region. Site III is located far downstream of the *SAV576* tsp (from positions +383 to +407) and overlaps the start codon of *SAV576* (Fig. 5C). This finding is analogous to a previous report that the binding site of JadR1 on the *cmlJ* promoter is far downstream of the *cmlJ* tsp, and that JadR1 negatively regulates *cmlJ* expression [44]. SAV576 may negatively regulate the transcription of its own gene by directly interfering with transcription elongation or by recruiting other repressors.

A palindromic sequence is a typical feature of TFR-binding targets. Analysis of the SAV576-binding sites using the DNAMAN program revealed that site II contains a perfect 15-bp palindromic sequence (termed “b”), and that sites I and III contain similar 15-bp sequences (termed “a” and “c”, respectively) (Fig. 5A and B). Sequences “a” and “c” have three mismatched nucleotides in comparison with “b”. The comparison of sequences “a”, “b” and “c” provided a consensus sequence, CCRTACRVYGTATGS (R: A or G; V: A, G, or C; Y: T or C; S: G or C) (Fig. 5D).

To estimate the relative contributions of the three 15-bp sequences to SAV576 protein binding, EMSAs were performed using a probe that contained either the intact 15-bp sequence or a mutated sequence (Fig. 5E). The affinity of SAV576 for the mutated probes (termed 1 m, 2 m, and 6 m), which lacked inverted repeats, was abolished completely in comparison with the corresponding wild-type 43-bp probes 1a, 2, and 6 (Fig. 5F). These results indicate that each of the three 15-bp sequences is important for SAV576-binding activity.

### *SAV575* Exerts a Positive Effect on Avermectin Production

**Figure 6:**
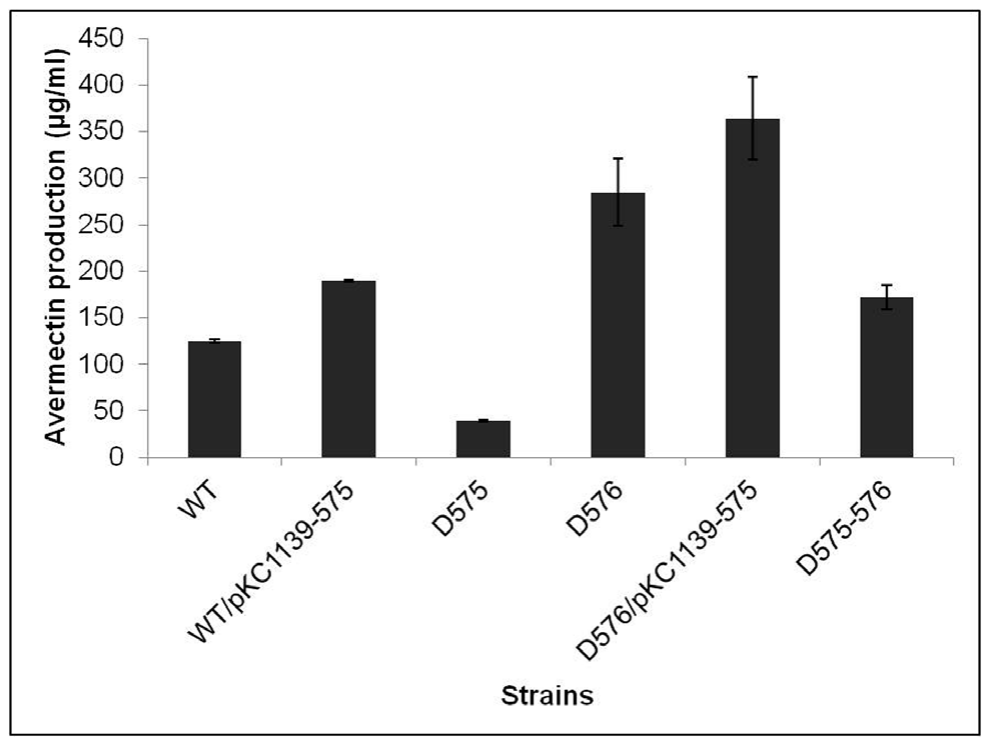
Comparison of avermectin production in *SAV575* mutant strains. The fermentation products were analyzed by HPLC following cultivation in FM-I for 10 days. WT/pKC1139-575, ATCC31267 containing *SAV575* overexpression vector; D575, *SAV575* mutant; D576, *SAV576* mutant; D576/pKC1139-575, D576 containing *SAV575* overexpression vector; D575-576, *SAV575-SAV576* double mutant.

Because *SAV575* was found to be a target gene of SAV576, we further investigated its relationship with avermectin production. A comparison of avermectin production among various *S. avermitilis* strains revealed that the overexpression of *SAV575* in wild-type strain (WT/pKC1139-575) and D576 (D576/pKC1139-575) increased avermectin production, whereas the deletion of *SAV575* in wild-type strain (D575) and D576 (D575-576) led to decreased avermectin production (Fig. 6). These results indicate that *SAV575* has a positive effect on avermectin production.

Because *SAV575* is a cytochrome P450 family gene, it may have no effect on gene expression. To test this hypothesis, we used semiquantitative RT-PCR to measure the transcription of *SAV576* and *ave* genes in ATCC31267 and D575. No marked difference in the transcription levels of these genes was observed (Fig. S4). These results imply that *SAV575* exerts its positive effect on avermectin production through some means other than the control of gene expression.

## Discussion

TFRs are the third most common transcriptional regulator family in bacteria and regulate a wide range of cellular activities, including antibiotic production, multidrug resistance, amino acid metabolism, osmotic stress, pathogenicity, and development [45]. However, many of the functions of TFRs remain poorly known or unknown. Some *Streptomyces* species, including *S. coelicolor* and *S. avermitilis*, contain over 100 TFR genes. This large number presumably reflects the complex morphological differentiation and secondary metabolism in these species. Of the 115 predicted TFRs in the *S. avermitilis* genome, only two, SAV3818 [31] and SAV3703 (AvaR3, a γ-butyrolactone-autoregulator receptor) [32] have been characterized as positive regulators of avermectin production. The findings of the present study demonstrate an important role of SAV576, a novel TFR, in the negative control of avermectin production in *S. avermitilis*. We are currently engaged in characterization of other differentially expressed regulatory genes revealed by transcriptome analysis.

The expression of *SAV576* was found to be autoregulated. However, the differing transcription levels of *SAV576* at various life stages indicate that the regulation of *SAV576* expression is complex and is controlled in part by other yet-unknown upstream factor(s). Studies using a bacterial one-hybrid system will help identify the transcriptional regulators that interact with the *SAV576* promoter region. Transcription and binding experiments have shown that SAV576 indirectly downregulates the expression of *aveR*, which encodes the pathway-specific activator for avermectin biosynthesis [24], [25]. It appears likely that SAV576 controls yet-unknown gene(s) that directly regulate *aveR* expression. The *aveR* promoter was shown to be directly recognized and activated by housekeeping Eσ^hrdB^ *in vitro* [46]. However, it remains unclear what types of transcriptional regulators directly control *aveR* expression in *S. avermitilis*. A transcriptional activator AtrA (SCO4118) was shown to directly regulate the transcription of *act*II-ORF4, the pathway-specific activator of the actinorhodin biosynthetic gene cluster in *S. coelicolor* [47]. However, AtrA and its homolog AveI (SAV4110) in *S. avermitilis* functioned as negative regulators for avermectin biosynthesis, and no direct binding of AtrA or AveI to the promoter region of *aveR* was observed [29]. Although the γ-butyrolactone-autoregulator receptor AvaR3 positively regulates *aveR* expression, there is no ARE sequence in the promoter region of *aveR*, suggesting that such regulation is indirect [32]. Deletion of *avaR3* did not affect the transcription level of the *adpA* homolog *SAV5261* [32]. We demonstrated previously that the *adpA* homolog in *S. avermitilis* is not involved in the regulation of avermectin production [41]. Taken together, these findings indicate that *S. avermitilis* has a novel, *adpA*-independent pathway for the regulation of avermectin biosynthesis. The identification of direct regulators of *aveR* is essential for better understanding of the regulatory network of avermectin biosynthesis.

Among the SAV576 target genes, we investigated *SAV575* in view of its predicted function. SAV575 (CYP102D1) is a member of the cytochrome P450 (CYP) family that catalyzes the monooxygenation of a variety of hydrophobic substrates and plays a key role in primary and secondary metabolic pathways and drug detoxification [48]. *S. avermitilis* contains 33 CYPs, whereas *S. coelicolor* contains only 18. Of the CYP genes in *S. avermitilis*, 11 are located within the secondary metabolite gene cluster and are predicted to be involved in secondary metabolite production [49]. Lamb *et* *al.* predicted that some CYP genes that are not linked to a specific gene cluster contribute to secondary metabolite production [49]. *SAV575* is not cluster-situated, and its product CYP102D1 is a unique self-sufficient P450 that has no homolog in *S. coelicolor*. *SAV575* or its orthologs in other Actinobacteria (SCAB5931 in *S. scabies*, SCLAV2750 in *S. clavuligerus*, SACE4205 in *Saccharopolyspora erythraea*) had no previously assigned function in the context of antibiotic biosynthesis; however, our findings indicate that *SAV575* is involved in avermectin production. The ability of CYP102D1 to catalyze the oxidation of saturated and unsaturated fatty acids [50] suggests that the function of SAV575 is to provide precursors, such as acetate and propionate extender units, for avermectin biosynthesis by oxidizing fatty acids or other compounds. The *SAV575* transcription level was very low in wild-type strain ATCC31267. Deletion of *SAV576* increased the *SAV575* transcription level, presumably increasing the availability of precursors for avermectin biosynthesis and thereby increasing avermectin production. Another possibility is that SAV575 produces molecules that bind to an activator of avermectin biosynthesis to stimulate its DNA-binding affinity or bind to a repressor to eliminate or reduce its DNA-binding affinity. We did not investigate the role of *SAV574*, another SAV576 target gene, in avermectin biosynthesis. *SAV574* encodes a dehydrogenase, and may indirectly provide energy or precursors for avermectin biosynthesis by catalyzing the degradation of certain substrates. Further studies are necessary to evaluate these possibilities and to clarify the function of *SAV574*.

EMSAs and footprinting assays revealed a 15-bp consensus sequence CCRTACRVYGTATGS that is important for SAV576-binding activity. Similar sequences were also found in many other promoter regions (Table S2). Further experiments will establish which of these genes are SAV576 targets, and will clarify the relationships of the newly discovered SAV576 target genes with *aveR* expression and avermectin biosynthesis. Improved knowledge of the SAV576 regulatory mechanism will lead to more effective strategies for increasing avermectin production.

## Supporting Information

Figure S1 **Transcription levels of** ***aveR, aveA1, SAV151, SAV576, SAV292, SAV880,*** **and** ***SAV4189*** **in avermectin-overproducing strain 76-02-e relative to those in wild-type strain ATCC31267.** Samples were collected from each strain grown in FM-II medium after days 2 and 6 of growth. *hrdB* was used as an internal control. Standard deviations are indicated by error bars (n = 3). Each gene was examined by relative quantification real-time RT-PCR with gene-specific primers.
(TIF)

Figure S2 **Confirmation of the transcriptional units** ***SAV574-SAV575*** **and** ***SAV579-SAV580-SAV581*** **by RT-PCR.** Lanes: RT, RT-PCR; –, negative control with reverse transcriptase omitted; +, positive control with genomic DNA of ATCC31267 as the template. The primers used for amplifying the *SAV574-SAV575* intergenic region were GJ109 (GCGCTACCAGCAGGACGT) and GJ110 (CTCCTCCACGGCGAACTT); those used for amplifying the *SAV579-SAV581* intergenic region were GJ159 (ACCGCACCCATCAGGAAG) and GJ160 (GGTAAGAAACGAGGGCGTA).
(TIF)

Figure S3 **Determination of the transcriptional start points of** ***SAV575*** **(A) and** ***SAV576*** **(B) by 5′-RACE PCR.** Boxed area: 5′-RACE oligo dT-anchor primer.
(TIF)

Figure S4 **Effect of** ***SAV575*** **deletion on the expression of related genes.** Semiquantitative RT-PCR analysis of transcription levels of genes from ATCC31267 (WT) and D575. The strains were grown in FM-II for 6 days. *hrdB* was used as a positive internal control.
(TIF)

Table S1 **Primers used in this study.**
(DOCX)

Table S2 **Putative targets of SAV576.**
(DOCX)

File S1 **List of genes having an expression level at least two-fold higher or lower in avermectin overproducer 76-02-e in comparison with wild-type strain ATCC31267.**
(XLS)
