# Supplementary figures and images for: A Novel TetR Family Transcriptional Regulator, SAV576, Negatively Controls Avermectin Biosynthesis in Streptomyces avermitilis
Source: PLoS One. 2013 Aug 13;8(8):e71330. doi: 10.1371/journal.pone.0071330 (PMC3742746; doi:10.1371/journal.pone.0071330)

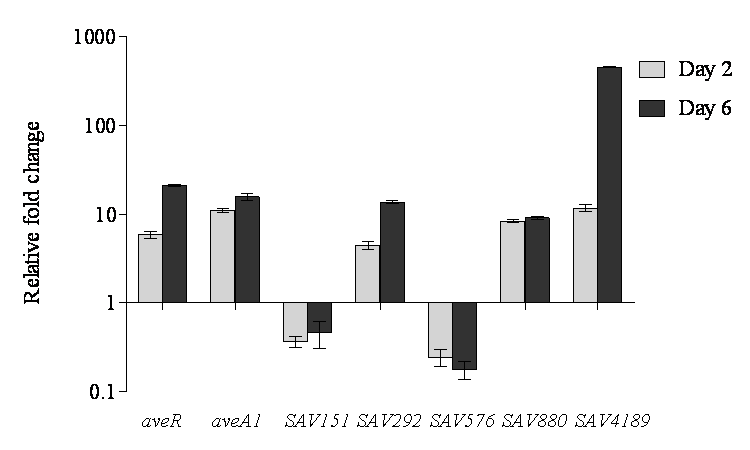

Supplement: Figure S1 — Transcription levels of aveR, aveA1, SAV151, SAV576, SAV292, SAV880, and SAV4189 in avermectin-overproducing strain 76-02-e relative to those in wild-type strain ATCC31267. Samples were collected from each strain grown in FM-II medium after days 2 and 6 of growth. hrdB was used as an internal control. Standard deviations are indicated by error bars (n = 3). Each gene was examined by relative quantification real-time RT-PCR with gene-specific primers. (TIF) [file pone.0071330.s001.tif]

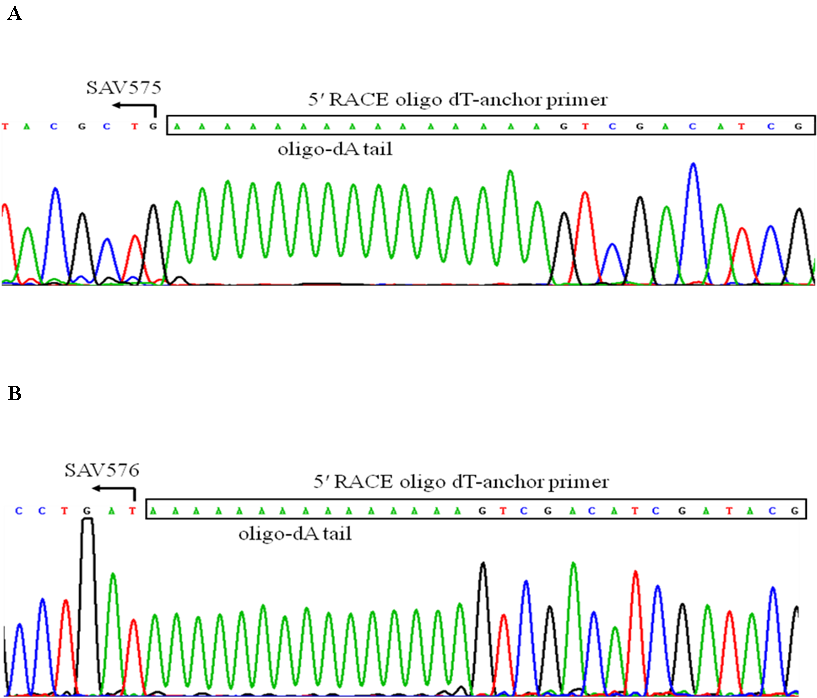

Supplement: Figure S3 — Determination of the transcriptional start points of SAV575 (A) and SAV576 (B) by 5′-RACE PCR. Boxed area: 5′-RACE oligo dT-anchor primer. (TIF) [file pone.0071330.s003.tif]
