# Supplementary material for: A Novel TetR Family Transcriptional Regulator, SAV576, Negatively Controls Avermectin Biosynthesis in Streptomyces avermitilis
Source: PLoS One. 2013 Aug 13;8(8):e71330. doi: 10.1371/journal.pone.0071330 (PMC3742746; doi:10.1371/journal.pone.0071330)
Supplement: Table S1 — Primers used in this study. (DOCX) [file pone.0071330.s005.docx]

**Table S1.** Primers used in this study.

| Primer | | DNA sequence (5'-3') | | Use | | |  |
| --- | --- | --- | --- | --- | --- | --- | --- |
| Primers for gene disruption, complementation, and overexpression | | | | | | |  |
| GJ45 | | CGGAATTCCTCAATCGGCAGACCTGG, *Eco*RI | | Delete *SAV576* gene | | |  |
| GJ46 | | GAAGATCTCTTCCGTTCAGTCGCCAT, *Bgl*II | |  |  |  |  |
| GJ47 | | GAAGATCTTGTCGCACGAGTCCGC, *Bgl*II | |  | | |  |
| GJ48 | | CCCAAGCTTGACGAACGAGGGCTTTGG, *Hin*dIII | |  | | |  |
| GJ51 | | ATGGCTGGCTCCTCCAAG | | Confirm *SAV576* deletion  mutant D576 | | |  |
| GJ52 | | TGATGATCGCCGAGCTC | |  |  |  |  |
| GJ55 | | CTGCCCGTCGCCAGTTAC | |  | | |  |
| GJ56 | | CGGTGACACAGGACAGGG | |  | | |  |
| GJ45 | | CGGAATTCCTCAATCGGCAGACCTGG, *Eco*RI | | Complement D576 | | |  |
| GJ56* | | GCTCTAGACGGTGACACAGGACAGGG, *Xba*I | |  | | |  |
| GJ125 | | CGGAATTCAAAGGCTCAGGCGGGAAG, *Eco*RI | | Delete *SAV575* gene | | |  |
| GJ126 | | GAAGATCTGGTCTCGGGCTGTGTGGT, *Bgl*II | |  | | |  |
| GJ127 | | GAAGATCTCAGGACGCCATTGTGGGT, *Bgl*II | |  | | |  |
| GJ128 | | CCCAAGCTTAACAGCGGGACGAGGGAG, *Hin*dIII | |  | | |  |
| GJ135 | | GCTTCCGTTCAGTCGCCATA | | Confirm *SAV575* deletion | | |  |
| GJ136 | | GGTGACCGTGTCGGAGGT | | mutant D575 | | |  |
| GJ79 | | GGGCGATGAAGGGCTACT | |  | | |  |
| GJ80 | | GGTCGTCCTCACCTGTGC | |  | | |  |
| GJ89 | | CGGAATTCCGGAAGCGGGCTGTGT, *Eco*RI | | Overexpress *SAV575* | | |  |
| GJ90 | | GCTCTAGATGCCGTGCGGTGTCTC, *Xba*I | | in *S. avermitilis* | | |  |
| GJ71 | | GGAATTTCATATGGCGACTGAACGGAAG, *Nde*I | | Overexpress His_6_-tagged | | |  |
| GJ72 | | GGAATTCGCCGACGAGAGACAAGC, *Eco*RI | | SAV576 protein *in E. coli* | | |  |
| Primers for ChIP assay | | | | | | |  |
| GJ105 | | GGTATTCCATTCGGTGTTGC | | Detect *aveR* promoter DNA | | |  |
| GJ106 | | TGTTATGAATTTGCCCTGGTG | |  |  |  |  |
| GJ107 | | CGGATCGTGGGTGTCCTC | | Detect *SAV575* and *SAV576* promoter DNA | | |  |
| GJ108 | | GAAACTCAATCGGCAGACCT | |  |  |  |  |
| B1n | | ACCCTCGGATCGTGC | | Detect *hrdB* promoter DNA | | |  |
| B2n | | AGCGCCATGACAGAGAC | |  |  |  |  |
| Primers for semiquantitative RT-PCR | | | | | | |  |
| GJ79 | | GGGCGATGAAGGGCTACT | | *SAV575* ORF | | |  |
| GJ80 | | GGTCGTCCTCACCTGTGC | |  |  |  |  |
| GJ83 | | GTCGGTTCGCAGTTCAGC | | *SAV576* ORF | | |  |
| GJ84 | | GAATCTCCTCGTCGGGCA | |  |  |  |  |
| aveR-S | | CCGCGACTTCCTCACCG | | *aveR* ORF | | |  |
| aveR-AS | | GGACTCGCTCAGCAG | |  |  |  |  |
| aveA1-S | | ACGCTTCCGACGTCTTCCG | | *aveA1* ORF | | |  |
| aveA1-AS | | TTGTCCTCGGTCCACGGAG | |  |  |  |  |
| hrdB-S | | TACTGCGCAGCCTCAACCAG | | *hrdB* ORF | | |  |
| hrdB-AS | | GCCGATCTGCTTGAGGTAGTC | |  |  |  |  |
| Primers for real-time RT-PCR | | | | | | |  |
| GJ111 | | CCACCTGGACAGCAAGAAGT | | | | *SAV574* ORF |  |
| GJ112 | | GGACATCCGAGAGGCAGAC | | | |  |  |
| GJ93 | | ATCTGCACCCCTTCCTCAAC | | | | *SAV575* ORF |  |
| GJ94 | | ACTTCTTGTCATCGGCCTTG | | | |  |  |
| GJ137 | | CCATCAAGGCCACCAGTTC | | | | *SAV576* ORF |  |
| GJ138 | | GTTTTCAGCCCCTCGTTCCT | | | |  |  |
| GJ46* | | CTTCCGTTCAGTCGCCAT | | | | *SAV576* non-coding region |  |
| GJ55* | | CTGCCCGTCGCCAGTTAC | | | |  |  |
| GJ97 | | CAGAAGAACTCACGCTCGTC | | | | *aveR* ORF |  |
| GJ98 | | ACTCTTTCCACAGCCCATTC | | | |  |  |
| GJ99 | | CGGACAGGACTACGCACTTC | | | | *aveA1* ORF |  |
| GJ100 | | ACGAGATACGACCGGAGATG | | | |  |  |
| GJ91 | | CCAAGGGCTACAAGTTCTCC | | | | *hrdB* ORF |  |
| GJ92 | | TTGATGACCTCGACCATGTG | | | |  |  |
| R151A | | GACTCGTAGAAGTAGCGCTCG | | | | *SAV151* ORF |  |
| R151B | | ATGACCGGCACACAGACC | | | |  |  |
| 292real11 | | GAGTGTCGATCCAGCCTTGT | | | | *SAV292* ORF |  |
| 292real22 | | CGCTGAGAAGGAGTTCGAGT | | | |  |  |
| R880A | | ATGCCCGACTCTCCGACC | | | | *SAV880* ORF |  |
| R880B | | AAGTGCGCGAAGAGGGTC | | | |  |  |
| 4189real11 | | GGTCTGCTCATCCAGCTCAT | | | | *SAV4189* ORF |  |
| 4189real22 | | GTGTACGGGTGGTCGGAAC | | | |  |  |
| Primers for EMSA | | | | | | |  |
| GJ209 | GAAGGCATACAGCGTATGGAAAC | | | | | probe 1 | |
| GJ210 | ATGGCTGGCTCCTCCAAG | | | | |  |  |
| GJ123 | CCCAGGTCTGCCGATTGAGTTTCC  ATACGTCGTATGGTACTAC | | | | | probe 2 | |
| GJ124 | GTAGTACCATACGACGTATGGAAA  CTCAATCGGCAGACCTGGG | | | | |  |  |
| GJ141 | ACCTCCGCGATGCGTACT | | | | | probe 3 | |
| GJ142 | GAAACTCAATCGGCAGACCT | | | | |  |  |
| GJ181 | ATATGCCCTGCCGAGGAG | | | | | probe 4 | |
| GJ182 | GGAGGACACCCACGATCC | | | | |  |  |
| GJ139 | AGGACGGTGGCATCGTTT | | | | | probe 5 | |
| GJ140 | GTTCGCCAGGGTCTCCTC | | | | |  |  |
| GJ219 | AGTCGCCATACGCTGTACGGTA  CCAGCGCCCGCGGCACTGCGG | | | | | probe 6 | |
| GJ220 | CCGCAGTGCCGCGGGCGCTGG  TACCGTACAGCGTATGGCGACT | | | | |  |  |
| GJ105 | GGTATTCCATTCGGTGTTGC | | | | | probe 7 | |
| GJ106 | TGTTATGAATTTGCCCTGGTG | | | | |  |  |
| JL6 | ATCATGGTCGGGAACCTCC | | | | | probe 8 | |
| JL7 | TCACCGCTAGGCAATGCTC | | | | |  |  |
| GJ213 | TGAAGGCATACAGCGTATGGAA  ACGGGGCGCTGCCGTACCGGC | | | | | probe 1a | |
| GJ214 | GCCGGTACGGCAGCGCCCCGTT  TCCATACGCTGTATGCCTTCA | | | | |  |  |
| GJ215 | TGAAGCGAATTCGAAGCTTGAA  ACGGGGCGCTGCCGTACCGGC | | | | | probe 1m | |
| GJ216 | GCCGGTACGGCAGCGCCCCGTT  TCAAGCTTCGAATTCGCTTCA | | | | |  |  |
| GJ161 | CCCAGGTCTGCCGATTGAGTTTC  GAATTCTAAGCTTGTACTAC | | | | | probe 2m | |
| GJ162 | GTAGTACAAGCTTAGAATTCGAA  ACTCAATCGGCAGACCTGGG | | | | |  |  |
| GJ221 | AGTCGCGAATTCCAAGCTTGTAC  CAGCGCCCGCGGCACTGCGG | | | | | probe 6m | |
| GJ222 | CCGCAGTGCCGCGGGCGCTGGT  ACAAGCTTGGAATTCGCGACT | | | | |  |  |
| hrdBP11 | CATCGTTGACCACCTATGACC | | | | | *hrdB* promoter | |
| hrdBP22 | CTCTCGGAACGTTGGAAAAC | | | | |  |  |
| Primers for DNase I footprinting assay | | | | | | |  |
| GJ77 | | | GAGACCCTGGCGAACC | | *SAV575-SAV576*  intergenetic region | |  |
| FAM-GJ78 | | | GGCTGGCTCCTCCAAG | |  |  |  |
| GJ227 | | | CAGGGCATATCTGCGATCC | |  |  |  |
| FAM-GJ228 | | | AGTTTGCGCATGGTGAGC | |  |  |  |
| Primers for 5' RACE | | | | | | |  |
| 576SP1 | | | GGTACGTCTCTCTTTCTTGTG | | | *SAV576* | |
| 576SP2 | | | AGTCGCCATACGCTGTACG | | |  |  |
| 576SP3 | | | GTTTTCAGCCCCTCGTTCCT | | |  |  |
| 575SP1 | | | AGTAGCCCTTCATCGCCC | | | *SAV575* | |
| 575SP2 | | | TTCCTCTTCGTGCTGGTG | | |  |  |
| 575SP3 | | | ACTGCTTGGACAGCTTGC | | |  |  |
