# Supplementary material for: A Novel TetR Family Transcriptional Regulator, SAV576, Negatively Controls Avermectin Biosynthesis in Streptomyces avermitilis
Source: PLoS One. 2013 Aug 13;8(8):e71330. doi: 10.1371/journal.pone.0071330 (PMC3742746; doi:10.1371/journal.pone.0071330)
Supplement: Table S2 — Putative targets of SAV576. (DOCX) [file pone.0071330.s006.docx]

**Table S2.** Putative targets of SAV576.

| # | GENE ID | FUNCTION |
| --- | --- | --- |
| 1 | *SAV559* | putative secreted protein |
| 2 | *SAV577* | putative TetR family transcriptional regulator |
| 3 | *SAV581* | putative secreted protein |
| 4 | *SAV2723*, *rocA* | putative delta-1-pyrroline-5-carboxylate dehydrogenase |
| 5 | *SAV2747* | putative phosphodiesterase |
| 6 | *SAV3087* | putative peptide ABC transporter ATP-binding protein |
| 7 | *SAV3636* | putative SyrP-like protein |
| 8 | *SAV3967*, *cys* | putative cysteinyl-tRNA synthetase |
| 9 | *SAV5104*, *ftsZ* | putative cell division ATP-binding protein |
| 10 | *SAV6384* | hypothetical protein |
| 11 | *SAV1282*  *SAV1283* | putative two-component system response regulator  putative membrane protein |
| 12 | *SAV3305*, *metB*  *SAV3306* | putative cystathionine gamma-synthase  putative secreted protein |
| 13 | *SAV3929*  *SAV3930* | putative oxidoreductase  putative secreted protein |
| 14 | *SAV4604*, *ssgB*  *SAV4605*, *minD2* | putative morphological differentiation-associated protein  putative septum site determining protein |
| 15 | *SAV6130*  *SAV6131* | putative isoleucyl-tRNA synthetase  putative DNA-binding protein |
| 16 | *SAV6420*  *SAV6421*, *fucA2* | putative lysozyme precursor, secreted  putative fuculose-1-phosphate aldolase |
| 17 | *SAV6457*  *SAV6458* | putative membrane protein  putative transglycosylase associated protein |
| 18 | *SAV7038*  *SAV7039* | putative siderophore binding protein  putative sugar acetyltransferase |

Numbers highlighted in yellow indicate that the 15-bp consensus sequence CCRTACRVYGTATGS was found in the bidirectional promoter regions of these divergently transcribed genes.
